# Association between dietary carotenoid intakes and abdominal aortic calcification in adults: National Health and Nutrition Examination Survey 2013–2014

**DOI:** 10.1186/s41043-024-00511-9

**Published:** 2024-02-01

**Authors:** Weidong Chen, Yuanqing Li, Min Li, Hai Li, Caifang Chen, Yanzhao Lin

**Affiliations:** 1 The Second Clinical Medical College of Guangzhou University of Chinese Medicine, No. 111, Dade Road, Yuexiu District, Guangzhou, 510120 China; 2 Department of Integrated Traditional Chinese and Western Medicine Nutrition of The Sixth Affiliated Hospital, School of Medicine, South China University of Technology, Foshan, 528225 China; 3 Department of Cardiology, The Key Laboratory of Advanced Interdisciplinary Studies Center, The First Affiliated Hospital of Guangzhou Medical University, Guangdong, 510120 China; 4 College & Hospital of Stomatology, Guangxi Medical University, Nanning, 530021 China

**Keywords:** Carotenoid intakes, Abdominal aortic calcification, National Health and Nutrition Examination Surveys

## Abstract

**Objective:**

Abdominal aortic calcification (AAC) is an important marker of subclinical atherosclerosis and a predictor of cardiovascular disease. This study aims to explore the association between carotenoid intakes and AAC.

**Methods:**

We included 2889 participants from the National Health and Nutrition Examination Survey (NHANES). Dietary carotenoid intakes were obtained through 24-h dietary recall interviews. Severe AAC was defined as a Kauppila score > 5. The main analysis utilizes logistic and restricted cubic spline models.

**Result:**

Severe AAC was detected in 378 (13.08%) participants. In fully adjusted models, the odds ratios (OR) with 95% confidence intervals (CI) of α-carotene, β-carotene, β-cryptoxanthin, lycopene, lutein with zeaxanthin and total carotenoid intakes for individuals with severe AAC were 0.53 (0.23–0.77), 0.39 (0.19–0.80), 0.18 (0.05–0.62), 0.40 (0.20–0.78), 0.53 (0.32–0.88) and 0.38 (0.18–0.77) in the highest versus lowest quartile intake, respectively. Dose–response analyses revealed that all of the carotenoids were associated with decreased risk of severe AAC in a nonlinear trend. Total carotenoid intakes of at least 100ug/kg/day were associated with decreased odds for severe AAC.

**Conclusion:**

α-carotene, β-carotene, β-cryptoxanthin, lycopene, lutein with zeaxanthin and total carotenoids were inversely associated with the risk of severe AAC in adults.

**Supplementary Information:**

The online version contains supplementary material available at 10.1186/s41043-024-00511-9.

## Introduction

Atherosclerosis, a chronic inflammatory disease of large arteries, is a leading cause of cardiovascular diseases [1, 2]. Atherosclerosis has become an epidemic due to population growth and aging, and increased prevalence of comorbidities such as obesity and diabetes among others [3–5]. The abdominal aorta is among the earliest sites to develop atherosclerosis and calcification, which is a good marker of subclinical atherosclerosis and a predictor of cardiovascular disease [6, 7]. The absence of AAC is highly effective at ruling out coronary artery disease [8, 9]. The prevalence of AAC increases with age, present in 22.4% of males and 16.4% of females age under 45 years and 100% of both males and females at 75 years and older [10]. Therefore, early detection of AAC is of great significance in the primary prevention of cardiovascular disease.

Unhealthy lifestyle and diet are important contributors to atherosclerosis. More intake of fruits, vegetables and seaweed reduces the risk of atherosclerosis [11–13]. Carotenoids are fat-soluble pigments with antioxidant properties that are naturally present in fruits, vegetables and seaweed [14]. The relationship between carotenoid intakes and hypertension [15], depression [16] and cancer [17] has been reported in epidemiological studies. Li demonstrated that at least 100 ug/kg per day intakes of carotenoids was associated with lower risk of hypertension [15]. Ge found a U-shaped dose–response relationships between both β-carotene and lutein with zeaxanthin intakes and the risk of depressive symptoms [16]. Previous studies have reported that high dosage of lycopene is associated with lower risk of prostate cancer [17] and AAC [18]. However, the association between carotenoid intakes and AAC has not been fully investigated.

In this study, using data from National Health and Nutrition Examination Survey (NHANES), we aimed to explore the relationship between carotenoid intakes and AAC.

## Methods

### Study population

The data on carotenoid intakes and AAC were obtained from NHANES 2013–2014 cohort. NHANES is a nationally representative survey, designed to assess the health and nutritional status of the civilian noninstitutionalized population in the 50 US states and the District of Columbia. The relevant information from the survey is publicly available at https://www.cdc.gov/nchs/nhanes/ [19].

**Fig. 1:**
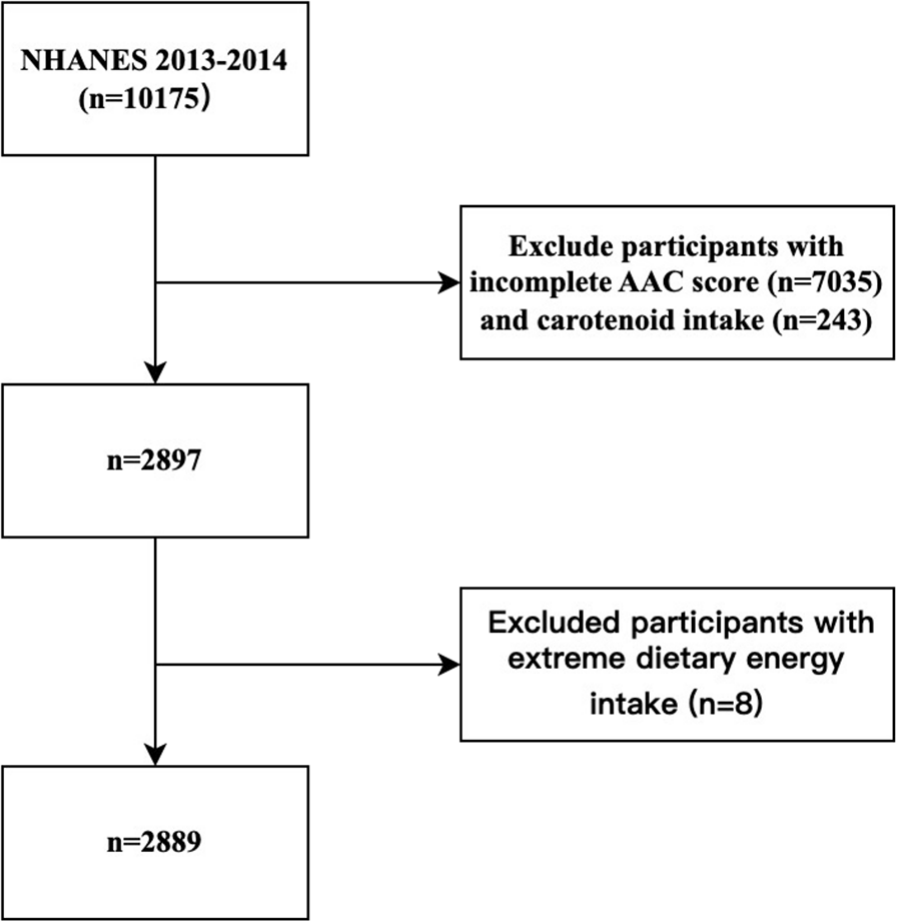
Flowchart of the eligible participants selection from NHANES 2013–2014

The NHANES 2013–2014 cohort included 10,175 participants. Participants with incomplete information on both carotenoid intakes (*n* = 7035) and AAC (*n* = 243) were excluded. Furthermore, we excluded participants with extreme dietary energy intake (male: < 500 or > 8000 kcal/day, female: < 500 or > 5000 kcal/day). The final analytical samples included 2889 individuals. Details are provided in Fig. 1.

### Dietary carotenoid intake assessment

NHANES database provides information on dietary carotenoids directly. The dietary carotenoid intakes of all included participants were obtained through two 24-h dietary recall interviews. The first diet recall interview was conducted in person at the Mobile Examination Center, and the second was conducted by telephone 3–10 days later. Each participant reports the type and amount of all food and beverages consumed 24 h prior to the interview. Multiple measurement guides (such as glasses, bowls, mugs, drink boxes, bottles, etc.) were used to estimate the amounts of food. Dietary data in NHANES were quantified by the Nutrition Methodology Working Group. In our study, the intake of α-carotene, β-carotene, β-cryptoxanthin, lycopene and lutein with zeaxanthin was calculated by averaging over the two recall periods. Total carotenoids were defined as the sum of the aforementioned five dietary carotenoids.

### AAC evaluation

AAC was assessed from lumbar lateral spine dual-energy X-ray absorptiometry scans (DXA, Densitometer Discovery A, Hologic, Marlborough, MA, USA). The Kauppila score was used to quantify the presence and severity of AAC. In the Kauppila score system, both anterior and posterior aortic walls were divided into four segments, corresponding to the region in front of the lumbar vertebrate L1–L4. The score 0 represents no calcification, 1 (≤ 1/3 of the aortic wall), 2 (> 1/3 to ≤ 2/3 of the aortic wall) or 3 (> 2/3 of the aortic wall) for these four segment. The Kauppila AAC score is the sum of calcification score, resulting in a range from “0” to “6” for each segment and “0” to “24” for the total score. According to previous studies, a higher AAC score corresponded to a more serious calcification condition of the abdominal aorta and severe AAC is defined as an AAC score > 5 [20].

### Covariates

Standard questionnaires were used to collect age, gender, race, family income, smoking status, physical activity and alcoholic intake. Race was classified as Hispanic, non-Hispanic white, non-Hispanic black or other race. Family monthly poverty level index were categorized as ≤ 1.30, 1.31 to 3.50 and > 3.50 (richest) [21]. Smoking status were categorized as current smoker, former smoker and never smoker based on whether they were currently smoking and smoked at least 100 cigarettes in his/her lifetime [22]. Alcohol drinking status were classified as none, moderate drinking (0–4 in males, 0–3 drinks/day in females) and heavy drinking (≥ 5 in males, ≥ 4 drinks/day in females) [23]. Metabolic equivalent (MET) was used to quantify physical activity. In NHANES, the MET for vigorous work or leisure-time activity was 8.0, moderate work or leisure-time activity 4.0, and walking or bicycling transportation 4.0, respectively. Participants were classified into inactive (< 600 MET-minutes/week)) and active (≥ 600 MET-minutes/week according to the physical activity guidelines for adults [24]. Energy intake data were obtained from two 24-h dietary recall and calculated as an average of 2-day energy intake. Body mass index (BMI) was calculated as weight in kilograms divided by height in meters squared. Hypertension was defined as if blood pressure ≥ 130/80 mmHg or taking antihypertensive medication [25]. Diabetes was defined as any of the following: taking insulin or diabetes pills, hemoglobin A1c ≥ 6.5% or fasting plasma glucose ≥ 126 mg/dl [26]. Dyslipidemia was defined as any of the following: total cholesterol ≥ 240 mg/dl, low-density lipoprotein cholesterol ≥ 160 mg/dl, high-density lipoprotein cholesterol < 40 mg/dl or currently taking cholesterol-lowering medication [27].

### Statistical analysis

Characteristics are described as means (SD) for continuous variables and frequency (percentages) for categorical variables. Differences between groups were tested using Student’s t-tests for continuous variables and Chi-squared tests for categorical variables. Carotenoid intakes were analyzed as a categorical variable (quartile). Logistic regression models were performed to estimate the association between carotenoid intakes and severe AAC. Model 1 included adjustment for age and gender. Model 2 included the variables in model 1 and race, family income, energy intake, BMI, smoking, alcohol drinking, physical activity, hypertension, diabetes and dyslipidemia. The lowest quartile (Q1) of carotenoid intakes was defined as the reference group. The dose–response analysis was conducted by restricted cubic spline model. In sensitivity analyses, ACC was evaluated as continuous variable (AAC score 0–24). Linear regression models were used to evaluate the association between dietary carotenoid intakes and ACC score. Interaction and stratified analyses were conducted according to age (< 60 or ≥ 60 years), gender (male or female), race (Hispanic white, non-Hispanic white, non-Hispanic black or other race), hypertension (yes or no), diabetes (yes or no) and dyslipidemia (yes or no). A *p* value < 0.05 was considered statistically significant. All tests were two-sided. All analyses were performed using SPSS 26.0 (IBM Inc., Chicago, IL, USA) and R 3.3.0 software.

## Results

The baseline characteristics of included population are presented in Table 1. The prevalence of severe AAC was observed in 13.08% participants. Overall, participants with severe AAC were more likely to be older, non-Hispanic White, and have lower BMI and lower energy intake. They were also more likely to be smokers and drinker and suffered from hypertension, diabetes and dyslipidemia.

**Table 1:** Characteristics of the study population

Characteristics	All (*N* = 2889)	Without severe AAC (*N* = 2511)	With severe AAC (*N* = 378)	*P*-value
Age	58.6 (11.9)	56.9 (11.2)	70.0 (10.1)	< 0 001
Male, *n*(%)	1391 (48.1%)	1211 (48.2%)	180 (47.6%)	0.868
Race, *n*(%)				< 0.001
Hispanic white	275 (9.52%)	250 (9.96%)	25 (6.61%)	
Non-Hispanic white	1303 (45.1%)	1075 (42.8%)	228 (60.3%)	
Non-Hispanic black	569 (19.7%)	510 (20.3%)	59 (15.6%)	
Other race	742 (25.7%)	676 (26.9%)	66 (17.5%)	
Ratio of family income to poverty:				0.053
< = 1.30	802 (30.0%)	694 (29.9%)	108 (30.3%)	
1.30–3.5	930 (34.8%)	792 (34.0%)	141 (39.6%)	
> = 3.5	942 (35.2%)	835 (36.0%)	107 (30.1%)	
BMI	28.5 (5.60)	28.7 (5.73)	27.3 (4.47)	< 0.001
Hypertension, n(%)	1735 (60.8%)	1434 (57.8%)	301 (80.3%)	< 0.001
Diabetes mellitus, n(%)	584 (20.6%)	456 (18.5%)	128 (34.3%)	< 0.001
Dyslipidemia, n(%)	1142 (40.6%)	913 (37.4%)	229 (61.7%)	< 0.001
Physical activity n(%)				0.001
Active	551 (26.2%)	467 (25.0%)	84 (35.4%)	
Inactive	1556 (73.8%)	1403 (75.0%)	153 (64.6%)	
Smoking status, n(%)				< 0.001
Never	1558 (53.9%)	1402 (55.9%)	156 (41.3%)	
Former	804 (27.8%)	664 (26.5%)	140 (37.0%)	
Current	526 (18.2%)	444 (17.7%)	82 (21.7%)	
Drinker, n(%)				< 0.001
None	130 (6.77%)	90 (5.38%)	40 (16.0%)	
Moderate	1592 (82.9%)	1406 (84.1%)	186 (75.2%)	
Heavy	198 (10.3%)	176 (10.5%)	22 (8.80%)	
Energy intake (kcal/day)	1967 (812)	1992 (820)	1803 (736)	< 0.001
α-Carotene intake (μg/kg per day)	1.23 (0.33, 6.86)	1.35 (0.37, 7.68)	0.65 (0.10, 2.43)	< 0.001
β-Carotene intake (μg/kg per day)	15.51 (5.72, 35.52)	17.18 (6.23, 41.97)	7.72 (3.29, 21.72)	< 0.001
β-cryptoxanthin intake (μg/kg per day)	0.58 (0.19, 1.36)	0.61 (0.19, 1.47)	0.39 (0.14, 0.87)	< 0.001
Lycopene intake (μg/kg per day)	27.99 (6.82, 76.06)	30.78 (8.21, 81.35)	10.88 (0.02, 35.62)	< 0.001
Lutein with zeaxanthin intake (μg/kg per day)	11.37 (6.16, 22.26)	11.71 (6.73, 22.50)	8.85 (4.11, 20.26)	0.012
Total carotene intake (μg/kg per day)	84.87 (41.93, 163.40)	92.70 (45.89, 175.68)	48.96 (25.71, 48.96)	< 0.001

*BMI* Body mass index

Table 2 presents the odds ratios (ORs) with 95% CIs of severe AAC based on quartiles of five carotenoids and total of them. The relationships between carotenoid intakes and severe AAC are shown in Table 2. The crude ORs with 95% CI of severe AAC in the highest versus lowest quartiles were 0.25 (0.17–0.35) for α-carotene, 0.31 (0.22–0.43) for β-carotene, 0.37 (0.26–0.54) for β-cryptoxanthin, 0.27 (0.19–0.38) for lycopene, 0.53 (0.39–0.71) for lutein with zeaxanthin and 0.18 (0.13–0.27) for total carotenoid. After adjustment for age and gender in Model 1, the results remained stable and statistically significant. In Model 2, the ORs with 95% CI of severe AAC were 0.53 (0.23–0.77) for α-carotene, 0.39 (0.19–0.80) for β-carotene, 0.18 (0.05–0.62) for β-cryptoxanthin, 0.40 (0.20–0.78) for lycopene, 0.53 (0.32–0.88) for lutein with zeaxanthin and 0.38 (0.18–0.77) for total carotenoid.

**Table 2:** Multivariable-adjusted odds ratios with 95% CIs of severe AAC based on quartiles of five carotenoids and total of them

	Cutoff μg/kg per day	Crude	Model 1	Model 2	*P* for trend^a^
		OR (95% CI)	OR (95% CI)	OR (95% CI)	
α-carotene intake					
Quartile 1	< 0.33	Ref.	Ref.	Ref.	0.028
Quartile 2	0.33 to < 1.23	0.50 (0.38–0.67)*	0.63 (0.46–0.87)*	0.63 (0.38–1.06)	
Quartile 3	1.23 to < 6.86	0.67 (0.50–0.88)*	0.62 (0.46–0.84)*	0.62 (0.37–1.02)*	
Quartile 4	at least 6.86	0.25 (0.17–0.35)*	0.35 (0.24–0.51)*	0.53 (0.23–0.77)*	
β-carotene intake					
Quartile 1	< 5.73	Ref.	Ref.	Ref.	0.028
Quartile 2	5.73 to < 15.51	0.57 (0.43–0.76)*	0.59 (0.44–0.80)*	0.68 (0.38–1.24)	
Quartile 3	15.51 to < 39.51	0.36 (0.26–0.50)*	0.47 (0.33–0.65)*	0.42 (0.21–0.82)*	
Quartile 4	at least 39.51	0.31 (0.22–0.43)*	0.55 (0.39–0.79)*	0.39 (0.19–0.80)*	
β-cryptoxanthin intake					
Quartile 1	< 0.19	Ref.	Ref.	Ref.	0.041
Quartile 2	0.19 to < 0.58	1.09 (0.82–1.45)	0.92 (0.72–1.34)	0.72 (0.40–1.27)	
Quartile 3	0.58 to < 1.36	0.91 (0.68–1.22)	0.88 (0.64–1.21)	0.59 (0.28–1.22)*	
Quartile 4	at least 1.36	0.37 (0.26–0.54)*	0.42 (0.28–0.61)*	0.18 (0.05–0.62)*	
Lycopene intake					
Quartile 1	< 6.81	Ref.	Ref.	Ref.	< 0.001
Quartile 2	6.81 to < 27.80	0.63 (0.48–0.83)*	0.89 (0.67–1.20)	0.73 (0.45–1.18)	
Quartile 3	27.80 to < 76.06	0.33 (0.24–0.45)*	0.58 (0.41–0.81)*	0.53 (0.30–0.94)*	
Quartile 4	at least 76.06	0.27 (0.19–0.38)*	0.49 (0.34–0.71)*	0.40 (0.20–0.78)*	
Lutein with zeaxanthin intake					
Quartile 1	< 6.16	Ref.	Ref.	Ref.	0.048
Quartile 2	6.16 to < 11.37	0.51 (0.38–0.69)*	0.65 (0.47–0.90)*	0.57 (0.33–1.01)	
Quartile 3	11.37 to < 22.26	0.45 (0.33–0.61)*	0.53 (0.38–0.73)*	0.57 (0.33–0.98)*	
Quartile 4	at least 22.26	0.53 (0.39–0.71)*	0.62 (0.45–0.85)*	0.53 (0.32–0.88)*	
Total carotene intake					
Quartile 1	< 41.93	Ref.	Ref.	Ref.	0.012
Quartile 2	41.93 to < 84.86	0.59 (0.45–0.78)*	0.75 (0.56–1.01)	1.01 (0.63–1.61)	
Quartile 3	84.86 to < 163.40	0.41 (0.31–0.55)*	0.64 (0.46–0.88)*	0.57 (0.33–1.01)	
Quartile 4	at least 163.40	0.18 (0.13–0.27)*	0.34 (0.23–0.51)*	0.38 (0.18–0.77)*	

OR indicates odds ratios. model 1: Adjusted for age, gender. Model 2: Model 1 + race, BMI, family income, smoking status, hypertension, diabetes and energy intake. BMI, Body mass index

P for trend^a^, a indicated *p* value for the model 2. P for trend was calculated while treating carotenoids as continuous variables. *P < 0.05

Dose–response relationship between carotenoid intakes and severe AAC is shown in Additional file 1: Fig. S1. The results showed that higher carotenoids were associated with decreased risk of severe AAC in a nonlinear trend (p for nonlinearity < 0.05). Sensitivity analyses showed that higher carotenoid intakes were significantly associated with AAC score in fully adjusted models (Additional file 1: Table S1). There was no evidence of effect modification by age, gender, race, hypertension diabetes and dyslipidemia for carotenoid intakes and severe AAC associations (all *p* interaction > 0.05).

## Discussion

In this study, using a representative national sample of US population aged 40 years and over, we found that dietary carotenoid intakes (including α-carotene, β-carotene, β-cryptoxanthin, lutein with zeaxanthin, lycopene, dietary carotenoid and total carotenoid) were inversely associated with severe AAC. The associations remained significant after adjusting for confounding variables. This suggested that carotenoid intakes play a protective role in the development of AAC. Subgroup analysis found that this association was independent of age, gender, race, hypertension, diabetes and dyslipidemia indicating that the associations were consistent in different population settings.

Previous studies have suggested that fruit and vegetable consumption have a great impact on calcification [28–30]. The CAIFOS (Calcium Intake Fracture Outcome Study) found that each 50-gram increase in apple intake per day results in 24% lower odds of developing severe AAC [(odd ratio OR): 0.76 (0.62 to 0.93), *p* = 0.009)]. In a previous NHANES study, every one standard deviation increase (9.4 g/day) in dietary fiber intake was associated with 28% lower risk of severe AAC [OR 0.72 (95% CI, 0.57 to 0.90), *p* = 0.004] [12]. Other studies have reported that the Mediterranean diet, which is rich in vegetables and fruits, negatively correlated with the risk of AAC [31, 32]. However, carotenoid has not been fully studied in previous research.

As carotenoids are widely found in fruits, vegetables and seaweed species, these studies indirectly suggest that carotenoid intakes reduce AAC risk. A population-based retrospective cohort study of the 6,095 chronic kidney disease participants demonstrated that higher intake of carotenoids associated with lower all-cause mortality. [α-carotene, (HR = 0.77, 95% CI 0.65 to 0.92, *P* = 0.002), β-cryptoxanthin (HR = 0.83, 95% CI 0.70 to 0.98, *P* = 0.019), lycopene (HR = 0.77, 95% CI 0.65 to 0.91, *P* = 0.002) and lutein + zeaxanthin (HR = 0.82, 95% CI 0.70 to 0.96, *P* = 0.002)] [33]. Our study showed that carotenoid intakes are inversely associated with severe AAC, and carotenoid intakes have a nonlinear negative dose–response relationship with severe AAC. Total carotenoid intakes of at least 100ug/kg/day were associated with decreased odds for severe AAC. The results of the present study are consistent with previous studies on fruit and vegetable consumption and AAC, suggested that carotenoid intakes may have a significant favorable impact on cardiovascular health.

The mechanisms behind the association between carotenoid intakes and AAC remain unclear. Several possible mechanisms which might explain this association have been proposed. First, it has been reported that inflammation caused by oxidative stress through reactive oxygen species (ROS) attack may play an important role in the pathophysiological process of vascular calcification [34–36]. Carotenoids are diet-derived antioxidants that can lower ROS activity, effectively protect endothelial cells from oxidation and cellular damages and consequently reduce the risk of developing AAC. Second, dietary carotenoid intakes were beneficial to lower blood pressure, which is an important risk factor for AAC [37]. Third, previous studies have indicated that IL-6 and TNF-α can promote vascular smooth muscle cell (VSMC) calcification and β-carotene can reduce the mRNA levels of IL-6 and TNF-α [38].

Interventions focused on reinstating nutrient levels in populations at nutritional risk may yield greater effectiveness compared to interventions concentrating on populations with sufficient nutrient levels and supplementing beyond adequacy [39]. This is particularly notable in light of the contradictory findings regarding the association of antioxidant intake or blood status, such as carotenoids, with chronic diseases or their risk factors, as well as the limited effects observed in antioxidant supplementary trials.

### Limitations of the study

This study had some limitations. First, the cross-sectional study cannot infer a causal relationship between carotenoid intakes and AAC. In addition, due to the cross-sectional design, reverse causation cannot be excluded. Second, although we adjusted for several potential confounders, there may still be some unknown confounding factors that cannot be ruled out. Third, the generalizability of our result may be limited because all participants were US residents aged 40 years and older in NHANES 2013–2014 due to the database limitations. Fourth, the complicated mechanism between carotenoid intakes and AAC warrants further investigation. Finally, carotenoid intake data were obtained from dietary recall interviews, which might have risk of recall bias.

## Conclusion

In conclusion, our study has found a negative and nonlinear association between carotenoid intakes and AAC in adults. This may provide a new strategy for preventing AAC, thereby reducing the risk of cardiovascular diseases. Further prospective studies are needed to confirm our results and elucidate the mechanisms involved.

### Supplementary Information

**Additional file 1. Fig S1.** The dose-response relationship between dietary carotenoid intakes and severe AAC. **Table S1.** Associations and Midlife Cognitive Function dietary carotenoid intakes and abdominal aortic calcification score.

## Data Availability

The data generated and analyzed during the current study are obtained from the NHANES 2013–2014 cohort. NHANES is a program conducted by the National Center for Health Statistics (NCHS), and the relevant information from the survey is publicly available at https://www.cdc.gov/nchs/nhanes/.

## Abbreviations

AAC: Abdominal aortic calcification
NHANES: National Health and Nutrition Examination Survey
OR: Odds ratios
CI: Confidence intervals
NCHS: National Center for Health Statistics
MET: Metabolic equivalent
BMI: Body mass index
ROS: Reactive oxygen species
VSMC: Vascular smooth muscle cell
