# Supplementary material for: Association between dietary carotenoid intakes and abdominal aortic calcification in adults: National Health and Nutrition Examination Survey 2013–2014
Source: J Health Popul Nutr. 2024 Feb 1;43:20. doi: 10.1186/s41043-024-00511-9 (PMC10835982; doi:10.1186/s41043-024-00511-9)
Supplement: Supplementary file 1 — Additional file 1. Fig S1. The dose-response relationship between dietary carotenoid intakes and severe AAC. Table S1. Associations and Midlife Cognitive Function dietary carotenoid intakes and abdominal aortic calcification score. [file 41043_2024_511_MOESM1_ESM.docx]

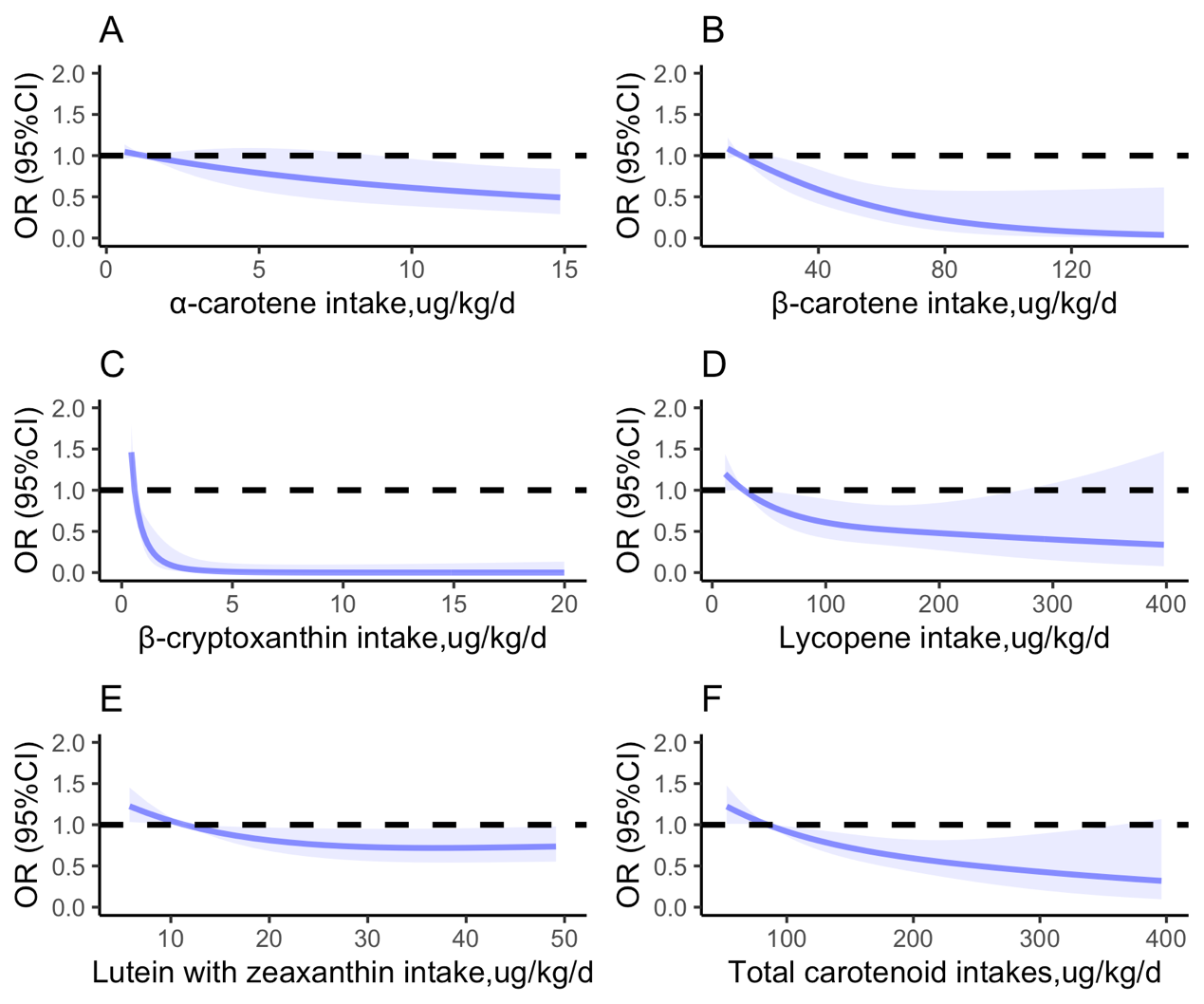


**FIGURE S1. The dose-response relationship between dietary carotenoid intakes and severe AAC.**

**Table S1. Associations between carotenoid intakes and abdominal aortic calcification score**

| **AAC score** | |
| --- | --- |
| Model 2 | |
| β (95% CI) | |
| α-carotene intake | -0.019 (-0.034, -0.004)* |
| β-carotene intake | -0.005 (-0.010, -0.001)* |
| β-cryptoxanthin intake | -0.029 (-0.054, -0.005)* |
| Lycopene intake | -0.002 (-0.004, -0.000)* |
| Lutein with zeaxanthin intake | -0.007 (-0.012, -0.001)* |
| Total carotene intake | -0.003 (-0.005, -0.002)* |

Model 2 included age, gender, race, family income, energy intake, BMI, smoking, alcohol drinking, physical activity, hypertension, diabetes and dyslipidemia. AAC, abdominal aortic calcification, BMI, Body mass index. * p < 0.05.
